# Smoking-attributable gastrointestinal cancer burden in Brazil, Russia, India, China and South Africa (BRICS) and Associated Economies: A Global Burden of Disease study analysis 1990**–**2023

**DOI:** 10.18332/tpc/221523

**Published:** 2026-07-13

**Authors:** Wei Jiang, Yinglin Wang, Rui Cheng

**Affiliations:** 1 Department of GastroenterologyBeijing Friendship Hospital, Capital Medical UniversityBeijingChina; 2 State Key Laboratory of Digestive HealthBeijingChina; 3 National Clinical Research Center for Digestive DiseaseBeijingChina; 4 Beijing Key Laboratory of Early Gastrointestinal Cancer Medicine and Medical DevicesBeijingChina

**Keywords:** gastrointestinal cancers, smoking, BRICS economies, mortality, disability-adjusted life years

## Abstract

**Introduction:**

BRICS and associated economies have a considerable share of the world’s population and cancer burden; however, long-term trends, heterogeneity and inflection points of smoking-attributable gastrointestinal (GI) cancers are still unclear. This retrospective population-based secondary analysis utilized Global Burden of Disease (GBD) 2023 data to investigate the mortality, disability-adjusted life years (DALYs) and age-standardized rates (ASRs) of smoking-attributable GI cancers among 11 BRICS and associated economies in 1990–2023, and to provide data-driven evidence for epidemiological and clinical prevention research on smoking-related GI cancers.

**Methods:**

This secondary analysis used 2023 GBD data, which included five main types of GI cancers caused by smoking: esophageal, stomach, colon and rectum, liver and pancreas cancers. Mortality, DALYs and ASRs were analyzed at the global, region and country levels. Joinpoint regression was used to compute annual percentage change (APC) and average annual percentage change (AAPC).

**Results:**

The total number of smoking-attributable GI cancer deaths in the study regions rose from 236305.08 in 1990 to 328324.75 in 2023 and total DALYs increased from 6.53 million to 8.08 million; the age-standardized mortality rate (ASMR) and age-standardized DALYs rate (ASDR) decreased with an AAPC of −1.44% and −1.48%, respectively. Brazil and China experienced the greatest declines in ASMR. India, Egypt and Ethiopia showed an increasing trend. Esophageal cancer was still the main cause of death and pancreatic cancer had the highest increase in mortality rate. Age-stratified analysis showed that the 20–29 years age group had a growing disease burden and the 40–64 years group had the largest decrease in DALYs. For people aged >75 years, the decline of disease burden decelerated.

**Conclusions:**

The burden of smoking-attributable GI cancers in BRICS and associated economies has increased with rising absolute mortality and DALYs and falling ASRs. Regional disparities, age trends and rising youth risks reveal marked epidemiological differences in GI cancer burden.

## Introduction

GI cancer is a major public health issue globally. Approximately 1 in 12 people will develop GI cancer during their life and 1 in 16 will die from it^1^. In 2022, the five major GI cancers – colorectal, gastric, liver, esophageal and pancreatic – made up 4.8 million new cases and 3.2 million deaths globally, accounting for 24% of all cancer cases and 33.3% of cancer-related deaths^2^. These cancers are the most common types of cancer in 27 countries and the main cause of cancer death in 36 countries^3^.

GI cancers have multiple modifiable risk factors and smoking is among the most important ones. In 2022, about 1.25 billion people aged ≥15 years were using tobacco products worldwide. From 1990 to 2021, smoking contributed to over 175 million deaths and almost 4.3 billion years of life were lost^4^. Smoking affects human health through multiple routes, such as tumor promotion. Cigarette smoke contains numerous harmful chemicals, more than 70 of which are known to cause cancer, these substances promote GI tumor formation through various mechanisms, including causing DNA damage, chronic inflammation and changing metabolic environment^5,6^. Understanding the epidemiological link between smoking and GI cancers is helpful for explaining the causes of diseases, formulating preventive measures and reducing the global cancer burden.

In order to address these gaps, the current research makes use of data from the GBD 2023 study to systematically analyze mortality, DALYs, and ASRs of smoking-attributable GI cancers in 11 BRICS and associated economies during a span of 33 years (from 1990 to 2023). We aim to determine the general and subtype-specific burden trends of smoking-related GI cancers and to provide data-driven evidence for understanding the epidemiological characteristics of these diseases.

## Methods

### Study design

This retrospective secondary analysis used open-access data from the GBD 2023 study to assess the burden of smoking-attributable GI cancers across 11 BRICS and associated economies from 1990–2023. We estimated mortality, DALYs and ASRs at global, regional and national levels. Joinpoint regression was used to quantify temporal trends via APC and AAPC. The study reveals an overall rise in absolute mortality and DALYs but a decline in ASRs, with substantial heterogeneity across countries, cancer subtypes and age groups.

### Data source

The GBD 2023 study evaluated health outcomes in 204 countries based on current medical research and advanced modeling techniques^7^. It identified 376 diseases and 88 risk factors that cause worldwide health harm from 1990 to 2023. Estimates are presented with 95% uncertainty intervals (UI), reflecting the possible range of actual values.

GI cancers were classified by using the following ICD-10 codes: esophageal (C15), gastric (C16), colorectal (C18-C21), liver (C22) and pancreatic (C25) cancers. We extracted data on mortality, DALYs, ASRs and years of life lost (YLL) smoking-attributable for each cancer type at global, regional and national levels.

### Age-standardized rate

ASRs were calculated by applying the GBD population standard. Final estimates are based on the average of 1000 model runs, where the 97.5th and 2.5th percentiles define the upper and lower bounds of the 95% UI^8^. The ASR was calculated as follows:

$$ASR=\frac{\sum_{i=1}^{A}a_{i}w_{i}}{\sum_{i=1}^{A}w_{i}}\times 100000$$

where A is the number of age groups, $a_{i}$ is the age-specific rate for age group i, $w_{i}$ the number of people in the corresponding age group i, among the standard population.

### Temporal trend analyses

Joinpoint regression was used to calculate the APC and AAPC in smoking-attributable GI cancer burden from 1990 to 2023^9^. AAPC was calculated as:

$$AAPC=\left\{exp\left(\frac{\sum w_{i}b_{i}}{\sum w_{i}}\right)-1\right\}\times 100$$

Where w_i_ indicates segment length and $b_{i}$ is the slope coefficient.

### Statistical analysis

Joinpoint regression was applied to calculate the APC and AAPC for temporal trends from 1990 to 2023. The trend was defined as increasing if the AAPC and its 95% confidence interval (CI) were both greater than 0, decreasing if both were less than 0 and stable otherwise^10^. A two-sided p<0.05 was considered statistically significant. All statistical analyses and data visualization were performed using R software (version 4.3.1). Packages including *dplyr* (version 1.1.3), *data.table* (version 1.17.0) and *ggplot2* (version 3.4.2) were used for data processing, transformation and graphical plotting.

## Results

### Overall burden of smoking-attributable gastrointestinal cancers

#### Mortality burden

The total number of smoking-attributable GI cancer deaths in BRICS countries showed a significant upward trend from 1990 to 2023. As presented in Table 1 and Supplementary file Table 1 the number of deaths has risen from 236305.08 (95% UI: 161390.63–322340.60) in 1990 to 328324.75 (95% UI: 233623.88–450831.41) in 2023. The AAMR increased from 43.87 (95% UI: 29.03–62.12) in 1990 to 47.76 (95% UI: 32.13–67.69) in 2023. In contrast, the ASMR fell from 67.78 (95% UI: 42.76–99.90) to 41.97 (95% UI: 26.72–61.84), with an AAPC of ‐1.44% (95% CI: ‐1.50–‐1.39).

**Table 1:** Mortality burden of smoking-attributable gastrointestinal cancers among BRICS and Associated Economies in 2023: Estimates from the Global Burden of Disease study

Cancers	Brazil	China	Egypt	Ethiopia	India	Indonesia	Iran	Russian Federation	Saudi Arabia	South Africa	UAE	Brics
**Gastrointestinal**												
**Number of deaths**	8672.34 (6243.52–11 477.86)	248628.03 (182343.50–334145.01)	3832.33 (1785.51–6419.89)	427.92 (204.70–790.42)	38393.44 (24888.24–56 604.14)	10243.32 (5419.83–17 056.43)	2195.73 (1296.63–3427.45)	13381.73 (9790.11–17 172.93)	284.02 (160.86–472.28)	2229.27 (1473.04–3199.75)	36.62 (17.93–65.25)	328324.75 (233623.88–450831.41)
**AAMR**	4.09 (2.94–5.41)	17.38 (12.75–23.36)	3.49 (1.63–5.85)	0.38 (0.18–0.70)	2.66 (1.72–3.92)	3.56 (1.89–5.93)	2.50 (1.47–3.90)	9.00 (6.59–11.55)	0.86 (0.49–1.43)	3.48 (2.30–4.99)	0.36 (0.18–0.64)	47.76 (32.13–67.69)
**ASMR**	3.25 (2.35–4.31)	10.61 (7.76–14.24)	5.69 (2.67–9.58)	0.84 (0.40–1.55)	3.22 (2.09–4.71)	3.60 (1.90–5.94)	2.75 (1.62–4.30)	5.18 (3.79–6.64)	1.86 (1.02–3.13)	4.06 (2.68–5.81)	0.91 (0.46–1.63)	41.97 (26.72–61.84)
**Esophageal**												
**Number of deaths**	3346.34 (2422.21–4400.08)	126485.42 (102275.00–154499.69)	282.07 (188.39–401.42)	156.69 (86.44–263.06)	28040.66 (19444.44–39 621.75)	2173.72 (1288.64–3489.74)	703.06 (445.95–1046.76)	3343.69 (2511.28–4132.86)	60.22 (35.56–99.85)	1138.69 (820.39–1526.23)	4.57 (2.61–7.88)	165735.13 (129520.92–209489.31)
**AAMR**	1.58 (1.14–2.07)	8.84 (7.15–10.80)	0.26 (0.17–0.37)	0.14 (0.08–0.23)	1.94 (1.35–2.75)	0.76 (0.45–1.21)	0.80 (0.51–1.19)	2.25 (1.69–2.78)	0.18 (0.11–0.30)	1.78 (1.28–2.38)	0.05 (0.03–0.08)	18.57 (13.95–24.16)
**ASMR**	1.25 (0.91–1.65)	5.36 (4.33–6.55)	0.46 (0.31–0.66)	0.31 (0.17–0.52)	2.38 (1.64–3.33)	0.77 (0.45–1.21)	0.92 (0.58–1.39)	1.28 (0.97–1.59)	0.47 (0.27–0.81)	2.11 (1.51–2.83)	0.17 (0.10–0.31)	15.50 (11.24–20.85)
**Stomach**												
**Number of deaths**	1778.11 (1406.82–2234.64)	62485.18 (43705.03–90 864.97)	664.02 (399.85–996.03)	85.62 (39.97–160.81)	4302.71 (2545.54–6627.47)	2015.46 (1228.85–3232.48)	772.69 (469.50–1175.70)	3515.74 (2722.44–4255.57)	58.00 (37.44–95.45)	211.78 (146.38–298.94)	7.88 (4.20–12.73)	75897.18 (52706.02–109954.79)
**AAMR**	0.84 (0.66–1.05)	4.37 (3.05–6.35)	0.60 (0.36–0.91)	0.08 (0.04–0.14)	0.30 (0.18–0.46)	0.70 (0.43–1.12)	0.88 (0.53–1.34)	2.37 (1.83–2.86)	0.18 (0.11–0.29)	0.33 (0.23–0.47)	0.08 (0.04–0.13)	10.71 (7.47–15.12)
**ASMR**	0.67 (0.53–0.85)	2.66 (1.86–3.84)	1.08 (0.65–1.64)	0.16 (0.07–0.31)	0.35 (0.21–0.54)	0.73 (0.43–1.17)	0.99 (0.60–1.48)	1.34 (1.04–1.62)	0.39 (0.24–0.63)	0.38 (0.26–0.54)	0.25 (0.13–0.42)	9.01 (6.03–13.05)
**Pancreatic**												
**Number of deaths**	1899.05 (1572.55–2312.16)	22974.05 (19019.81–27 976.86)	756.93 (535.21–1049.47)	38.30 (22.43–66.37)	2155.74 (1417.92–3030.04)	1729.16 (1106.78–2404.63)	348.27 (226.02–502.28)	3574.98 (3031.16–4320.65)	77.73 (53.99–107.60)	382.64 (299.65–495.68)	9.86 (5.73–15.33)	33946.71 (27291.26–42 281.08)
**AAMR**	0.90 (0.74–1.09)	1.61 (1.33–1.96)	0.69 (0.49–0.96)	0.03 (0.02–0.06)	0.15 (0.10–0.21)	0.60 (0.39–0.84)	0.40 (0.26–0.57)	2.41 (2.04–2.91)	0.24 (0.16–0.33)	0.60 (0.47–0.77)	0.10 (0.06–0.15)	7.71 (6.05–9.84)
**ASMR**	0.71 (0.59–0.87)	0.98 (0.81–1.19)	1.09 (0.76–1.51)	0.08 (0.04–0.13)	0.17 (0.12–0.25)	0.60 (0.38–0.84)	0.41 (0.26–0.60)	1.40 (1.19–1.70)	0.46 (0.30–0.65)	0.69 (0.54–0.90)	0.20 (0.12–0.31)	6.79 (5.11–8.94)
**Liver**												
**Number of deaths**	449.40 (143.65–795.21)	20649.31 (7023.70–35 867.13)	1814.74 (488.73–3462.21)	55.20 (12.72–127.07)	2321.34 (608.51–4807.02)	2295.98 (676.71–4693.81)	176.88 (47.39–391.45)	813.12 (255.54–1339.35)	53.45 (14.97–109.50)	295.01 (87.14–577.25)	7.16 (1.79–16.57)	28931.58 (9360.85–52 186.57)
**AAMR**	0.21 (0.07–0.37)	1.44 (0.49–2.51)	1.65 (0.45–3.15)	0.05 (0.01–0.11)	0.16 (0.04–0.33)	0.80 (0.24–1.63)	0.20 (0.05–0.45)	0.55 (0.17–0.90)	0.16 (0.05–0.33)	0.46 (0.14–0.90)	0.07 (0.02–0.16)	5.76 (1.72–10.86)
**ASMR**	0.17 (0.05–0.30)	0.91 (0.31–1.59)	2.59 (0.70–5.02)	0.10 (0.02–0.24)	0.18 (0.05–0.37)	0.77 (0.23–1.57)	0.21 (0.06–0.46)	0.32 (0.10–0.53)	0.34 (0.10–0.71)	0.51 (0.15–1.00)	0.15 (0.04–0.34)	6.26 (1.81–12.12)
**Colorectal**												
**Number of deaths**	1199.46 (698.29–1735.76)	16034.07 (10319.96–24 936.35)	314.57 (173.33–510.76)	92.12 (43.13–173.11)	1572.99 (871.82–2517.86)	2029.01 (1118.86–3235.77)	194.83 (107.76–311.27)	2134.21 (1269.69–3124.50)	34.62 (18.90–59.89)	201.15 (119.48–301.65)	7.15 (3.60–12.74)	23814.17 (14744.82–36 919.67)
**AAMR**	0.57 (0.33–0.82)	1.12 (0.72–1.74)	0.29 (0.16–0.47)	0.08 (0.04–0.15)	0.11 (0.06–0.17)	0.71 (0.39–1.13)	0.22 (0.12–0.35)	1.44 (0.85–2.10)	0.10 (0.06–0.18)	0.31 (0.19–0.47)	0.07 (0.04–0.13)	5.02 (2.95–7.71)
**ASMR**	0.45 (0.26–0.65)	0.69 (0.45–1.07)	0.46 (0.25–0.75)	0.19 (0.09–0.35)	0.13 (0.07–0.21)	0.72 (0.40–1.15)	0.23 (0.13–0.37)	0.83 (0.49–1.21)	0.19 (0.10–0.32)	0.36 (0.22–0.54)	0.15 (0.08–0.26)	4.40 (2.54–6.88)

ASMR: age-standardized mortality rate per 100000. AAMR: all ages mortality rate per 100000. Values in parentheses: 95% uncertainty intervals (UI) for corresponding estimates. UAE: United Arab Emirates.

Notably, esophageal and gastric cancers were still the main cause of death among GI cancers. The AAMR of esophageal cancer rose from 18.25 (95% UI: 12.83–23.86) in 1990 to 18.57 (95% UI: 13.95–24.16) in 2023, while that of gastric cancer decreased from 14.51 (95% UI: 10.13–20.68) to 10.71 (95% UI: 7.47–15.12). As for proportional share, esophageal cancer accounted for the largest share of total smoking-attributable GI cancer deaths (49.9% in 1990 and 50.5% in 2023), followed by gastric cancer (32.3% in 1990 and 23.1% in 2023). Pancreatic cancer showed the fastest growth in the proportion of deaths, increasing from 5.9% in 1990–10.3% in 2023.

#### DALYs burden

Consistent with mortality trends, the total DALYs attributable to smoking-related GI cancers in BRICS countries increased from 6527542.93 (95% UI: 4461600.02–8921955.71) in 1990 to 8081822.07 (95% UI: 5681851.61–11179844.50) in 2023 (Table 2 and Supplementary file Table 2) . The AADR slightly increased from 1233.27 (95% UI: 817.19–1746.89) to 1250.48 (95% UI: 829.82–1790.08), whereas the ASDR decreased from 1752.36 (95% UI: 1114.03,‐2565.83) to 1060.49 (95% UI: 672.93–1566.36), with an AAPC of ‐1.48% (95% CI: ‐1.53% to ‐1.42%).

**Table 2:** DALYs burden of smoking-attributable gastrointestinal cancers among BRICS and Associated Economies in 2023: Estimates from the Global Burden of Disease study

Cancers	Brazil	China	Egypt	Ethiopia	India	Indonesia	Iran	Russian Federation	Saudi Arabia	South Africa	UAE	BRICS
**Gastrointestinal**												
**Number of DALYs**	212547.03 (152627.42–279022.17)	5962387.40 (4335122.39–8040197.53)	105530.48 (48761.18–177806.19)	13819.30 (6532.53–25 445.79)	979393.95 (626852.80–1463664.56)	315782.88 (166098.29–530033.52)	58294.04 (34562.36–90 980.09)	359235.58 (263314.81–461899.56)	8874.76 (5057.29–14 926.66)	64796.99 (42362.25–93 797.38)	1159.66 (560.29–2071.05)	8081822.07 (5681851.61–11179844.50)
**AADR**	100.22 (71.97–131.57)	416.76 (303.02–562.00)	96.13 (44.42–161.96)	12.18 (5.76–22.44)	67.85 (43.43–101.40)	109.85 (57.78–184.38)	66.30 (39.31–103.47)	241.69 (177.15– 310.76)	26.90 (15.33–45.25)	101.14 (66.12–146.41)	11.45 (5.53–20.44)	1250.48 (829.82–1790.08)
**ASDR**	78.82 (56.61–103.57)	254.83 (184.32–343.56)	138.07 (64.36–232.17)	24.95 (11.90–46.01)	75.49 (48.77–111.94)	102.63 (54.09–171.28)	65.87 (39.07–102.88)	144.26 (105.66– 185.37)	45.64 (25.55–75.90)	112.11 (73.66–161.89)	17.82 (8.94–31.79)	1060.49 (672.93–1566.36)
**Esophageal**												
**Number of DALYs**	81214.85 (58641.72–106322.59)	2876968.19 (2330045.44–3450281.60)	7193.77 (4803.46–10 258.86)	4956.27 (2672.25–8245.04)	708881.40 (484756.98–1016314.85)	64923.07 (38718.53–107470.46)	17271.60 (11213.39–25 300.97)	89285.79 (67397.86–110884.94)	1682.56 (1004.10–2972.17)	31898.93 (22830.02–43 299.26)	127.29 (70.91–217.24)	3884403.73 (3022154.66–4881567.99)
**AADR**	38.30 (27.65–50.13)	201.10 (162.87–241.17)	6.55 (4.38–9.34)	4.37 (2.36–7.27)	49.11 (33.58–70.41)	22.58 (13.47–37.39)	19.64 (12.75–28.78)	60.07 (45.34– 74.60)	5.10 (3.04–9.01)	49.79 (35.64–67.59)	1.26 (0.70–2.14)	457.87 (341.78–597.83)
**ASDR**	30.05 (21.68–39.38)	120.83 (97.40–144.42)	10.13 (6.75–14.39)	9.13 (5.02–15.24)	54.87 (37.82–77.97)	21.31 (12.65–34.67)	20.34 (13.03–29.97)	35.47 (26.81– 44.08)	10.36 (6.10–17.43)	56.13 (40.23–75.99)	2.86 (1.65–5.09)	371.49 (269.14–498.64)
**Stomach**												
**Number of DALYs**	41449.20 (32597.28–51 822.20)	1450568.07 (1041458.89–2154775.19)	17221.33 (10265.48–25 963.83)	2881.44 (1384.44–5329.60)	110769.19 (66903.61–170660.29)	60765.16 (37680.40–95 867.53)	19864.56 (12184.98–30 339.16)	89627.92 (69048.07–108606.19)	1762.87 (1133.30–2924.84)	6303.03 (4392.84–8984.60)	235.14 (122.49–389.99)	1801447.92 (1277171.78–2655663.43)
**AADR**	19.54 (15.37–24.44)	101.39 (72.80–150.62)	15.69 (9.35–23.65)	2.54 (1.22–4.70)	7.67 (4.64–11.82)	21.14 (13.11–33.35)	22.59 (13.86–34.51)	60.30 (46.45– 73.07)	5.34 (3.44–8.87)	9.84 (6.86–14.02)	2.32 (1.21–3.85)	268.37 (188.30–382.89)
**ASDR**	15.48 (12.18–19.35)	61.61 (44.51–91.44)	23.98 (14.41–36.17)	5.01 (2.34–9.45)	8.49 (5.18–13.05)	20.05 (12.32–32.00)	22.80 (14.01–34.88)	35.39 (27.33– 42.80)	9.46 (5.99–15.49)	10.78 (7.48–15.25)	4.46 (2.37–7.43)	217.50 (148.11–317.31)
**Pancreatic**												
**Number of DALYs**	47208.81 (39614.36–56 147.20)	588251.11 (486392.77–711388.01)	21165.29 (14915.99–29 407.29)	1228.71 (721.06–2132.82)	55483.48 (36537.14–77 346.21)	53373.23 (33986.16–74 912.41)	10059.31 (6534.31–14 276.89)	99215.54 (84622.50–120067.52)	2526.20 (1782.54–3432.47)	11083.00 (8712.73–14 051.48)	323.56 (189.14–500.07)	889918.24 (714008.70–1103662.35)
**AADR**	22.26 (18.68–26.48)	41.12 (34.00–49.72)	19.28 (13.59–26.79)	1.08 (0.64–1.88)	3.84 (2.53–5.36)	18.57 (11.82–26.06)	11.44 (7.43–16.24)	66.75 (56.93– 80.78)	7.66 (5.40–10.41)	17.30 (13.60–21.93)	3.19 (1.87–4.94)	212.49 (166.49–270.58)
**ASDR**	17.46 (14.66–20.82)	25.39 (21.03–30.73)	27.24 (19.23–37.50)	2.23 (1.30–3.87)	4.24 (2.81–5.91)	17.28 (11.03–24.10)	10.86 (7.08–15.49)	40.53 (34.40– 48.88)	12.05 (8.18–16.57)	19.21 (15.14–24.46)	4.17 (2.48–6.53)	180.66 (137.34–234.86)
**Liver**												
**Number of DALYs**	11234.01 (3599.28–19 845.47)	621829.65 (212355.86–1070335.35)	50594.50 (13496.58–97 134.65)	1876.81 (433.82–4344.87)	63999.48 (16652.69–134380.27)	74139.73 (21926.53–152102.14)	5098.44 (1346.13–11 407.14)	22961.97 (7309.41– 37 664.14)	1693.79 (465.72–3475.76)	9525.90 (2785.41–18 611.62)	231.33 (57.50–530.00)	863185.60 (280428.93–1549831.40)
**AADR**	5.30 (1.70–9.36)	43.46 (14.84–74.81)	46.09 (12.29–88.48)	1.65 (0.38–3.83)	4.43 (1.15–9.31)	25.79 (7.63–52.91)	5.80 (1.53–12.97)	15.45 (4.92–25.34)	5.13 (1.41–10.54)	14.87 (4.35–29.05)	2.28 (0.57–5.23)	170.26 (50.77–321.84)
**ASDR**	4.16 (1.33–7.34)	28.38 (9.67–48.43)	64.92 (17.42–124.99)	3.24 (0.75–7.48)	4.78 (1.25–9.98)	23.51 (6.92–47.95)	5.52 (1.46–12.26)	9.53 (3.04–15.63)	8.44 (2.42–17.45)	15.72 (4.61–30.88)	3.11 (0.78–7.25)	171.30 (49.64–329.65)
**Colorectal**												
**Number of DALYs**	31440.16 (18174.79–44 884.70)	424770.38 (264869.44–653417.38)	9355.58 (5279.66–15 041.54)	2876.07 (1320.96–5393.47)	40260.39 (22002.38–64 962.95)	62581.68 (33786.67–99 680.98)	6000.13 (3283.54–9655.94)	58144.36 (34936.97– 84 676.77)	1209.35 (671.63–2121.42)	5986.12 (3641.25–8850.42)	242.34 (120.25–433.76)	642866.57 (388087.55–989119.33)
**AADR**	14.82 (8.57–21.16)	29.69 (18.51–45.67)	8.52 (4.81–13.70)	2.54 (1.16–4.76)	2.79 (1.52–4.50)	21.77 (11.75–34.68)	6.82 (3.73–10.98)	39.12 (23.50– 56.97)	3.67 (2.04–6.43)	9.34 (5.68–13.81)	2.39 (1.19–4.28)	141.48 (82.48–216.95)
**ASDR**	11.67 (6.75–16.68)	18.63 (11.72–28.53)	11.79 (6.55–19.12)	5.34 (2.49–9.98)	3.11 (1.71–5.02)	20.48 (11.17–32.56)	6.36 (3.49–10.28)	23.34 (14.07– 33.97)	5.34 (2.86–8.96)	10.27 (6.20–15.31)	3.22 (1.67–5.49)	119.55 (68.70–185.90)

DALYs: disability-adjusted life years. ASDR: age-standardized DALYs rate per 100000. AADR: all ages DALYs rate per 100000. Values in parentheses: 95% uncertainty intervals (UI) for corresponding estimates. UAE: United Arab Emirates.

Among specific cancer types, esophageal cancer was the leading contributor to DALYs; its AADR decreased from 496.14 (95% UI: 351.27–647.29) in 1990 to 457.87 (95% UI: 341.78–597.83) in 2023. In comparison, the ASDR of gastric cancer dropped from 402.72 (95% UI: 283.64–570.98) to 268.37 (95% UI: 188.30–382.89). YLL made up more than 90% of total DALYs for all cancer types.

### National-level burden variations

#### Mortality trends by country

**Figure 1:**
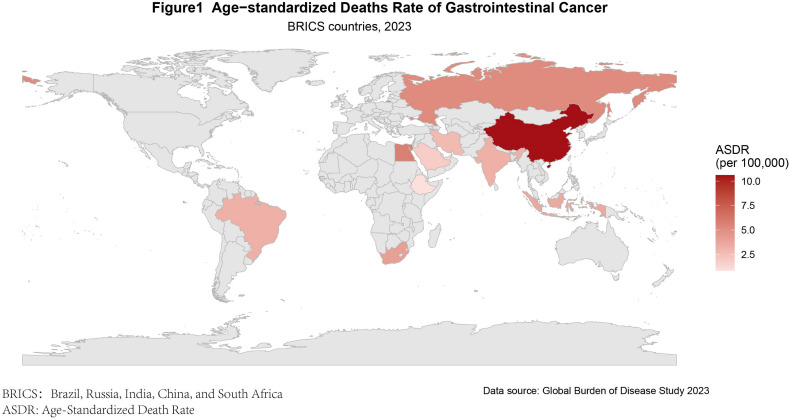
Age-standradized deaths rate of gastrointestoinal cancer

Marked heterogeneities in ASMR trends were observed across BRICS countries (Table 1 and Figure 1; and Supplementary file Table 1 and Figure 1). Brazil and China had the greatest declines in ASMR: Brazil’s ASMR dropped from 7.44 (95% UI: 5.59–9.42) in 1990 to 3.25 (95% UI: 2.35–4.31) in 2023 (AAPC=‐2.46%, 95% CI: ‐2.55–‐2.39), while China’s ASMR declined from 23.05 (95% UI: 15.82–31.36) to 10.61 (95% UI: 7.76–14.24) (AAPC=‐2.26%, 95% CI: ‐2.36%–‐2.15%). South Africa (AAPC= ‐2.05%, 95% CI: ‐2.14–‐1.95) and the Russian Federation (AAPC= ‐1.21%, 95% CI: ‐1.33–‐1.08) also showed declining trends. In contrast, India’s ASMR slightly increased from 2.65 (95% UI: 1.57–3.95) to 3.22 (95% UI: 2.09–4.71) (AAPC=0.53%, 95% CI: 0.41–0.63).

At the cancer subtype level (Supplementary file Figure 2), Brazil’s gastric cancer (AAPC= ‐4.03%, 95% CI: ‐4.12–‐3.97) and esophageal cancer (AAPC= ‐2.65%, 95% CI: ‐2.73–‐2.58) had the steepest mortality declines. China also saw rapid reductions in gastric cancer (AAPC= ‐2.98%, 95% CI: ‐3.08–‐2.87) and esophageal cancer (AAPC= ‐2.34%, 95% CI: ‐2.45–‐2.22). India was the only BRICS country with an increasing trend in esophageal cancer mortality (AAPC=1.65%; 95% CI: 1.54–1.74), while its gastric cancer mortality decreased (AAPC= ‐2.25%; 95% CI: ‐2.39–‐2.11). Pancreatic cancer mortality grew most rapidly in non-BRICS countries included in the analysis, such as Egypt (AAPC=3.82%; 95% CI: 3.64–3.98), Ethiopia (AAPC=2.81%; 95% CI: 2.73–2.89) and Saudi Arabia (AAPC=1.77%; 95% CI: 1.67–1.85).

Notably, China’s esophageal cancer ASMR in 2023 (5.36; 95% UI: 4.33–6.55) was 4.29 times higher than Brazil’s (1.25; 95% UI: 0.91–1.65), making it the highest-burden country for this subtype among BRICS. Brazil’s gastric cancer ASMR declined by 74.2% (from 2.60; 95% UI: 2.01–3.25 to 0.67; 95% UI: 0.53–0.85), the largest reduction among all BRICS countries.

#### DALYs trends by country

**Figure 2:**
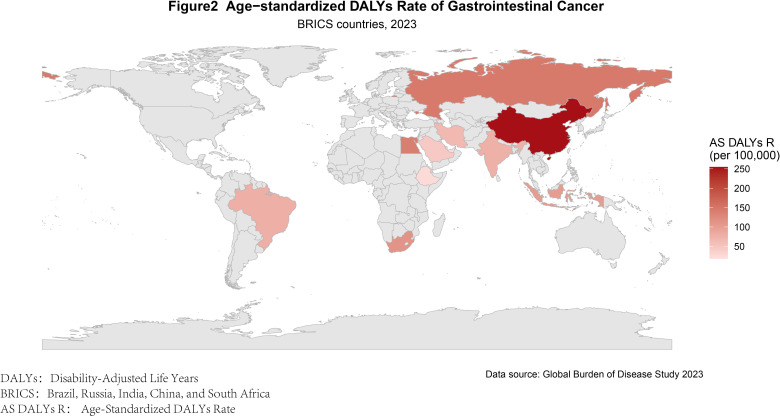
Age-standradized DALYs rate of gastrointestoinal cancer

**Figure 3:**
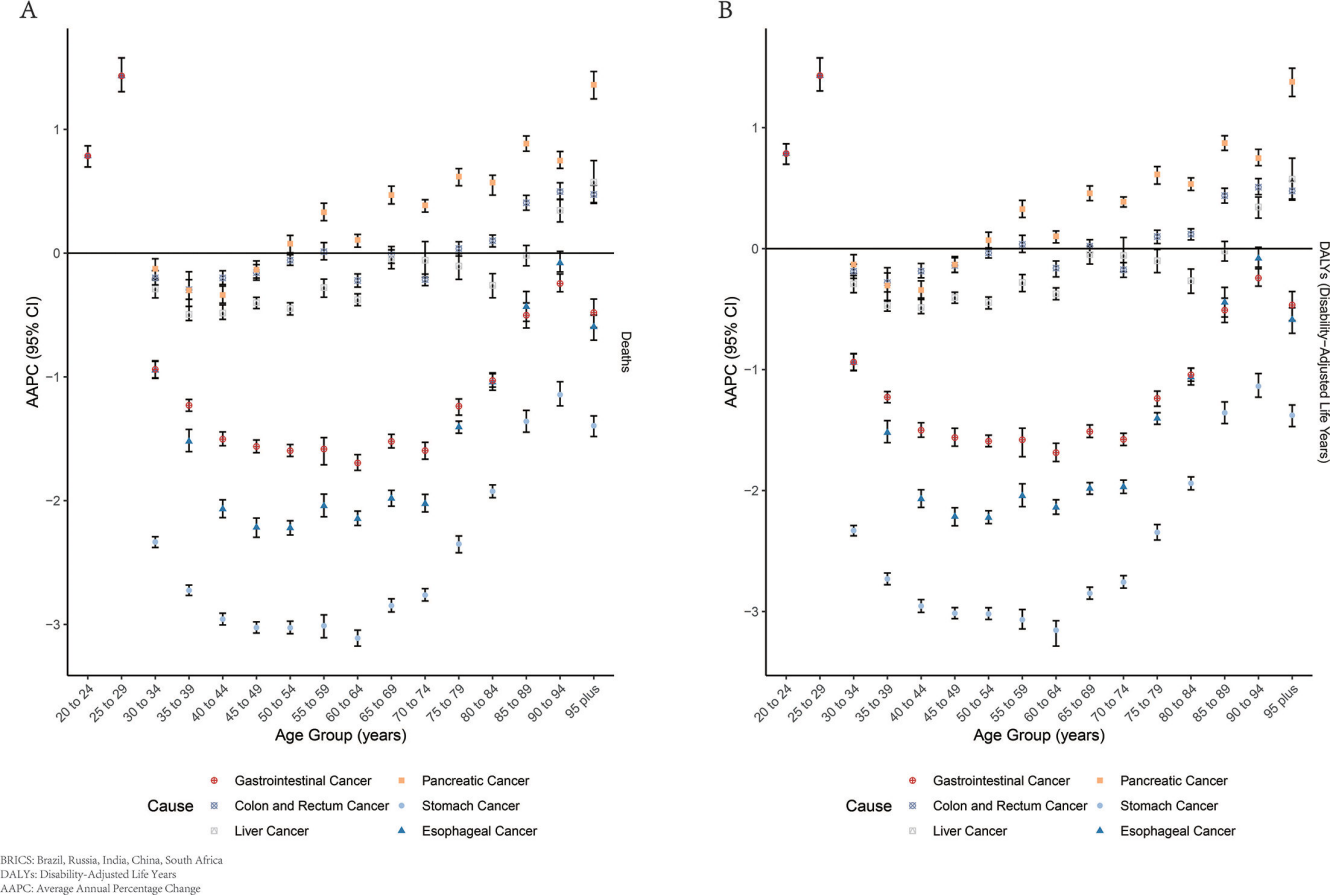
Age-stratified trends in mortality and DALYs of gastrointestinal-related cancers in BRICS

The ASDR trends by country were consistent with mortality trends (Table 2 and Figure 2; and Supplementary file Table 2 and Figure 3). Brazil’s ASDR plummeted from 185.23 (95% UI: 139.88–232.44) in 1990 to 78.82 (95% UI: 56.61–103.57) in 2023 (AAPC= ‐2.57%; 95% CI: ‐2.6–‐2.51), while China’s ASDR decreased from 591.16 (95% UI: 405.51–802.16) to 254.83 (95% UI: 184.32–343.56) (AAPC= ‐2.47%, 95% CI: ‐2.58–‐2.36). India slightly increased (AAPC=0.52%; 95% CI: 0.36–0.68) and the Russian Federation (AAPC= ‐1.34%; 95% CI: ‐1.42–‐1.25) and South Africa (AAPC=‐1.84%, 95% CI: ‐1.95–‐1.74) showed moderate declines.

For cancer subtypes (Supplementary file Figure 4), Brazil and China had the most significant reductions in gastric cancer (AAPC= ‐4.15%; 95% CI: ‐4.24–‐4.08 and ‐3.26%, 95% CI: ‐3.38–‐3.15 respectively) and esophageal cancer (AAPC= ‐2.76%, 95% CI: ‐2.82–‐2.7 and ‐2.67%, 95% CI: ‐2.78–‐2.55 respectively). India’s esophageal cancer DALYs burden kept increasing (AAPC=1.43%; 95% CI: 1.26–1.58). The ASDR of pancreatic cancer increased dramatically in Egypt (AAPC=3. 56%; 95% CI: 3.43–3.68) and Ethiopia (AAPC=2.74%; 95% CI: 2.67–2.8) and colorectal cancer rose in Egypt (AAPC=2.00%; 95% CI: 1.87–2.11), Ethiopia (AAPC=2.14%; 95% CI: 2.06–2.21) and Saudi Arabia (AAPC=1.57%; 95% CI: 1.47–1.66).

Joinpoint regression analysis found key inflection points in the trends. For example, China’s gastric cancer DALYs showed an accelerated decline during 2007–2014 (APC= ‐5.26%; 95% CI: ‐6.12–‐4.38) compared to 1990–2007 (APC= ‐2.99%; 95% CI: ‐3.51 – ‐2.47%). During 1999–2004, Egypt’s pancreatic cancer DALYs increased most rapidly (APC=10.19%; 95% CI: 8.23–12.20).

### Age-stratified trends

The age distribution of smoking-attributable GI cancer burden was highly heterogeneous (Figure 3). The age group of 20–29 years had positive AAPCs (DALYs: 0.79–1.43; mortality: 0.79–1.43). After 30 years, AAPCs became negative, with the lowest value appearing in the age group of 40–64 years (DALYs: ‐1.50–‐1.69; mortality: ‐1.50–‐1.70). For those >75 years, the declining trend slowed significantly and some subtypes even reversed to increasing.

Subtype-specific age trends were different; esophageal and gastric cancers showed consistent declines in all groups ≥30 years, gastric cancer had the largest decrease (AAPC <−2.73% in middle-aged groups). In contrast, pancreatic cancer had positive AAPCs from 50 years onwards, which increased with age, peaking in the ≥95 years group (DALYs: 1.38; mortality: 1.36). Liver cancer showed slight negative growth in middle-aged groups, but it turned into positive growth for those who were aged ≥90 years. Colorectal cancer had a minor decline in middle age but rose after 75 years, with a significant increase in the ≥90 years group (AAPC ≈ 0.50%).

It should also be noted that some high-risk ‘age-cancer’ combinations appeared: the 20–29 years group had the fastest growth in GI cancer burden (AAPC=0.79–1.43%), the 40–64 years group experienced the greatest decrease in gastric cancer burden (AAPC= -3.03%) and the ≥95 years group had the highest AAPC for colorectal cancer (0.47%). AAPC estimates for DALYs and mortality were nearly identical across age groups and subtypes.

### Uncertainty analysis

The 95% UI for mortality and DALYs estimates differed between countries and cancer types. India’s esophageal cancer ASMR UI was wider in 2023 (1.64–3.33) than in 1990 (0.88–1.88). In contrast, Brazil’s stomach cancer ASMR had a narrow UI (1990: 2.01–3.25; 2023 :  0.53–0.85).

## Discussion

This study systematically analyzed the burden of smoking-attributable GI cancers in 11 BRICS and associated economies during a period of 33 years (1990–2023). The number of deaths and DALYs caused by these cancers has increased greatly. In contrast, ASRs decreased sharply; Brazil, China and South Africa had the fastest ASR decreases, whereas India, Egypt and Ethiopia experienced increases. Esophageal and gastric cancers decreased consistently among all age groups >30 years, with gastric cancer showing the largest reduction. Pancreatic and colorectal cancers rose among adults ≥75 years, with pancreatic cancer hitting a peak in the ≥95 years group. The 40–64 years age group benefited most from interventions, with DALYs and AAPCs decreasing; 20–29 years group showed some early signs of growing burden, pointing to accumulating risks in younger people. YLL accounted for over 90% of total DALYs, indicating that the burden was predominantly due to premature mortality. Distinct age-related patterns were observed, with rising burden in young adults and marked reductioThe cross-country differencesns in middle-aged groups. Mortality was the primary driver of burden changes. Wider UI in India suggested greater data variability, while narrower UI in Brazil indicated more reliable estimates; overall UI narrowed over time, reflecting improved data quality.

The latest global cancer statistics show that GI cancers are still the main cause of incidence and mortality around the world, but ASRs have remained stable or decreased in many areas^2^. The ‘growing total burden but declining rates’ trend found in this study is highly consistent with the general results of the GBD study. The decreasing ASR trends in our smoking-related study correspond to the findings from the GBD 2023 study, which accounts for all risk factors. It stated that the global ASRs for gastric cancer, esophageal cancer and colorectal cancer all decreased notably, while the number of related deaths increased substantially over the study period^11^. These different trends are closely related to factors such as population aging, screening popularization, *Helicobacter pylori* infection control and advances in prevention measures^11^.

Cross-country differences in trends show the uneven world spread of the GI cancer burden. ASR declines in Brazil, China and South Africa are due to great progress in tobacco control^12^ and focused cancer screening programs^12^. In contrast, rising ASRs in India, Egypt and Ethiopia are linked to changing tobacco epidemic patterns, ongoing risk factors and limited access to healthcare systems, highlighting regional differences in worldwide cancer management^2^. For heterogeneity across cancer types and age groups, the Sharma and Rakshit^13^ ecological study has shown that smoking has different effects on different types of cancer, the ASR of tobacco-related esophageal and gastric cancer burdens shows a downward trend, but the number of tobacco-related pancreatic and colorectal cancer cases increases globally. It is worth noting that the early warning signs of a growing burden in the 20–29 years group are similar to the Reitsma et al.^14^ findings on tobacco use among young people from 204 countries and regions, indicating that tobacco epidemics continue to be serious in certain areas and populations, and smoking behaviors among teenagers and young adults will increase the global cancer burden in the future^14^. Moreover, there is also a considerable rise in pancreatic cancer burden for people aged >95 years, this can be explained by the long latency effect of smoking^15^, nicotine and its derivatives promote pancreatic cancer via the HIF1A/YAP1 (Hypoxia-inducible factor 1 subunit alpha/ Yes1-associated transcriptional regulator) positive feedback loop^16^ and the current increase might be due to the smoking peak decades ago in the elderly.

The cross-country differences in ASR trends suggest the distinct disparities in smoking exposure patterns and cancer screening practice backgrounds across regions. Brazil and China followed the WHO Framework Convention on Tobacco Control (FCTC) recommendations such as imposing high taxes on tobacco products, adding graphic health warnings to cigarette packs and implementing comprehensive smoke-free public places regulations. These measures led to a decrease in smoking rates^17,18^. Targeted endoscopic screening programs that were encouraged in East Asia helped increase the rate of early detection and lowered the mortality rate for gastric and esophageal cancers^12,19^. In contrast, countries such as India and Ethiopia have inadequate tobacco control measures, poor enforcement and disparities in healthcare resource allocation, resulting in high late-stage diagnosis rates, poor prognosis and elevated mortality associated with tobacco-related diseases^20,21^.

The paradox of rising total deaths and DALYs along with falling ASRs is mainly caused by population aging^8^. In terms of GBD demographic information, there is a persistent and considerable rise in the percentage of individuals who are aged ≥65 years within the BRICS group and associated economies. GI cancer incidence increases rapidly as age increases; even if the age-specific incidence rate decreases, the total burden still experiences ‘mechanical’ growth because of the aging population^11^. Declining ASR shows that the risk factor intervention works and that the diagnosis and treatment have improved to counteract the effect of aging^11^. Multiple risk factors acting together make it hard to control GI cancer. Smoking, alcohol consumption, hypercholesterolemia, obesity and insufficient physical activity significantly increase disease risk^22^. For example, smoking and alcohol consumption acting together significantly increase the risk of GI cancer, particularly that of esophageal and pancreatic cancer^23^, which is why the burden of certain GI cancers has not decreased even though tobacco control has made progress in some countries. Rising risk signals among young people indicate that the younger generation faces many different types of newly emerging lifestyle-related risks^22^.

### Strengths and limitations

This study has several strengths. First, it uses the most recent GBD 2023 data, which allows for a 33 year long-term trend observation with strong data timeliness and completeness. Secondly, as it includes 11 BRICS and associated economies, it provides a comprehensive regional perspective on the burden of smoking-attributable gastrointestinal cancers. Lastly, Joinpoint regression models have been used to find the trend inflection points and the effect of the population’s age structure has been removed by using ASRs, making sure that the method is up to the standards of high-level research^24^.

Despite these strengths, this study also has certain limitations. First, GBD data are based on models and thus countries that have poor epidemiological surveillance systems will have large UI, leading to inaccurate estimations^18^. Second, limited by the GBD framework, there was no analysis of gender differences and details such as the amount of cigarettes smoked per day, smoking duration and whether they had quit were not included, so it was impossible to explore dose-response relationships^25,26^. Third, this study only adopted ecological analysis to explore the connection between relevant factors and disease burden changes, lacking individual-level data and quasi-experimental designs. Future research could incorporate improved tobacco surveillance data, individual cohort studies and more robust evaluations to better understand the actual impact of these measures.

## Conclusions

Smoking continues to be a major modifiable risk factor for GI cancers in BRICS and associated economies. The increasing total burden, regional disparities, different ages and cancer types and new risks appearing in young populations constitute a complex control landscape^4^. Further well-designed population-based longitudinal studies are still needed. They should clarify how tobacco exposure patterns, cancer detection strategies and multifactorial risk factors jointly shape the future burden of smoking-related GI cancers.

## Abbreviations

ASMR: age-standardized mortality rate per 100000
AAMR: all ages mortality rate per 100000
DALYs: disability-adjusted life years
ASDR: age-standardized DALYs rate per 100000
AADR: all ages DALYs rate per 100000
ASR: age-standardized rate per 100000
YLL: years of life lost
APC: annual percentage change
AAPC: average annual percentage change
