# Supplementary material for: Smoking-attributable gastrointestinal cancer burden in Brazil, Russia, India, China and South Africa (BRICS) and Associated Economies: A Global Burden of Disease study analysis 1990–2023
Source: Tob Prev Cessat. 2026 Jul 13;12:10.18332/tpc/221523. doi: 10.18332/tpc/221523 (PMC13365772; doi:10.18332/tpc/221523)
Supplement: Supplementary file Material 1 [file TPC-12-38-s001.pdf]

TOBACCO PREVENTION AND CESSATION

Supplementary file

© 2026 Jiang W. et al.

DOI: 10.18332/tpc/221523

The content has been provided by the author(s) and has not been reviewed, verified, or endorsed by European Publishing. It may not have undergone peer review. The views, opinions, and recommendations expressed are solely those of the author(s) and do not necessarily reflect the position of European Publishing. European Publishing accepts no responsibility or liability for any consequences arising from the use of, or reliance on, this content.

Supplementary file Figure 1 Disparities of Age-Standardized Death Rates of Gastrointestinal Cancers in BRICS Countries (2023)

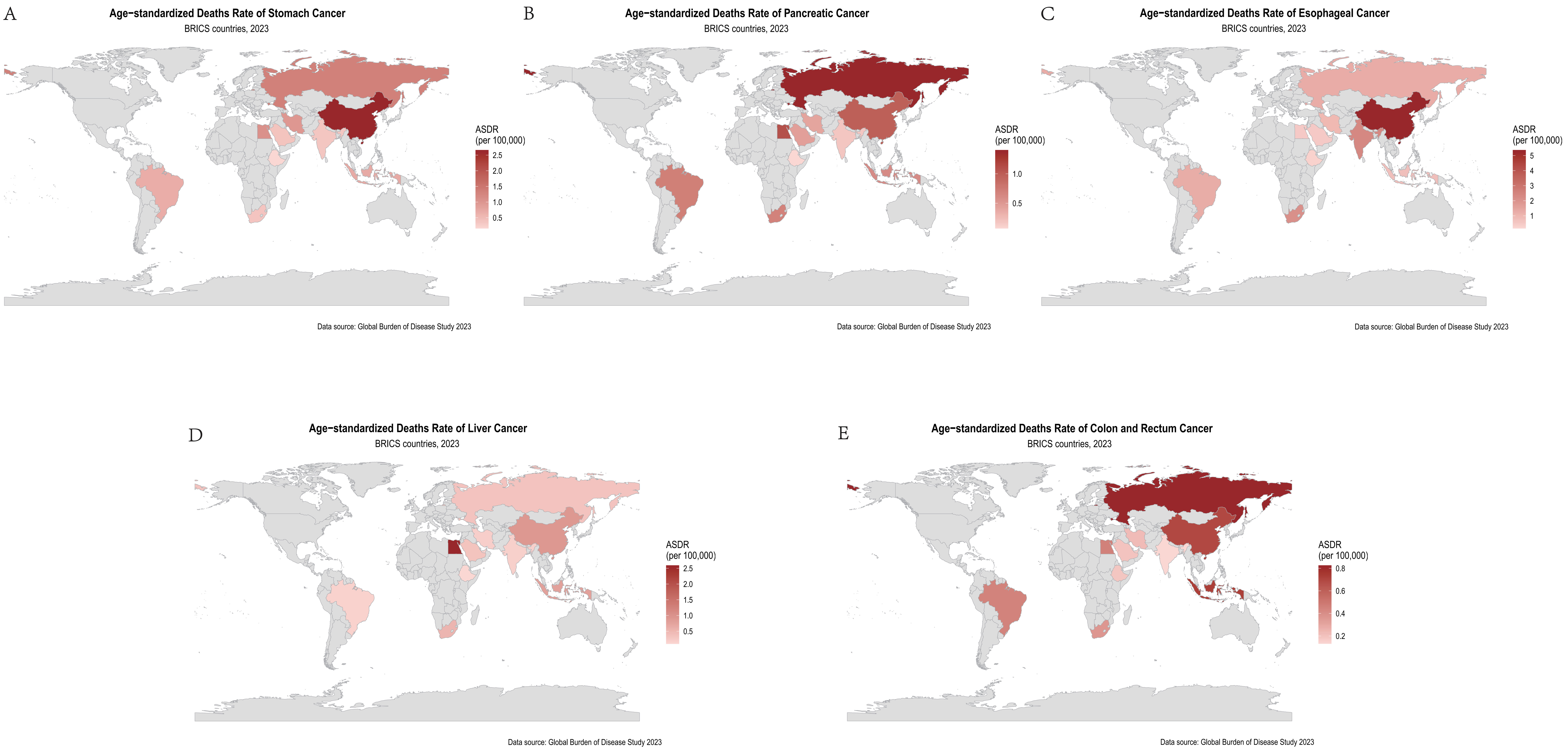

BRICS： Brazil, Russia, India, China, and South Africa
ASDR: Age-Standardized Death Rate
Cancer types: stomach cancer, pancreatic cancer, esophageal cancer,liver cancer, and colorectal cancer are the five main gastrointestinal cancer subtypes attributable to smoking.

Supplementary file Figure 2 Tobacco-Related Gastrointestinal Cancer Mortality Dynamics in BRICS (1990-2023)

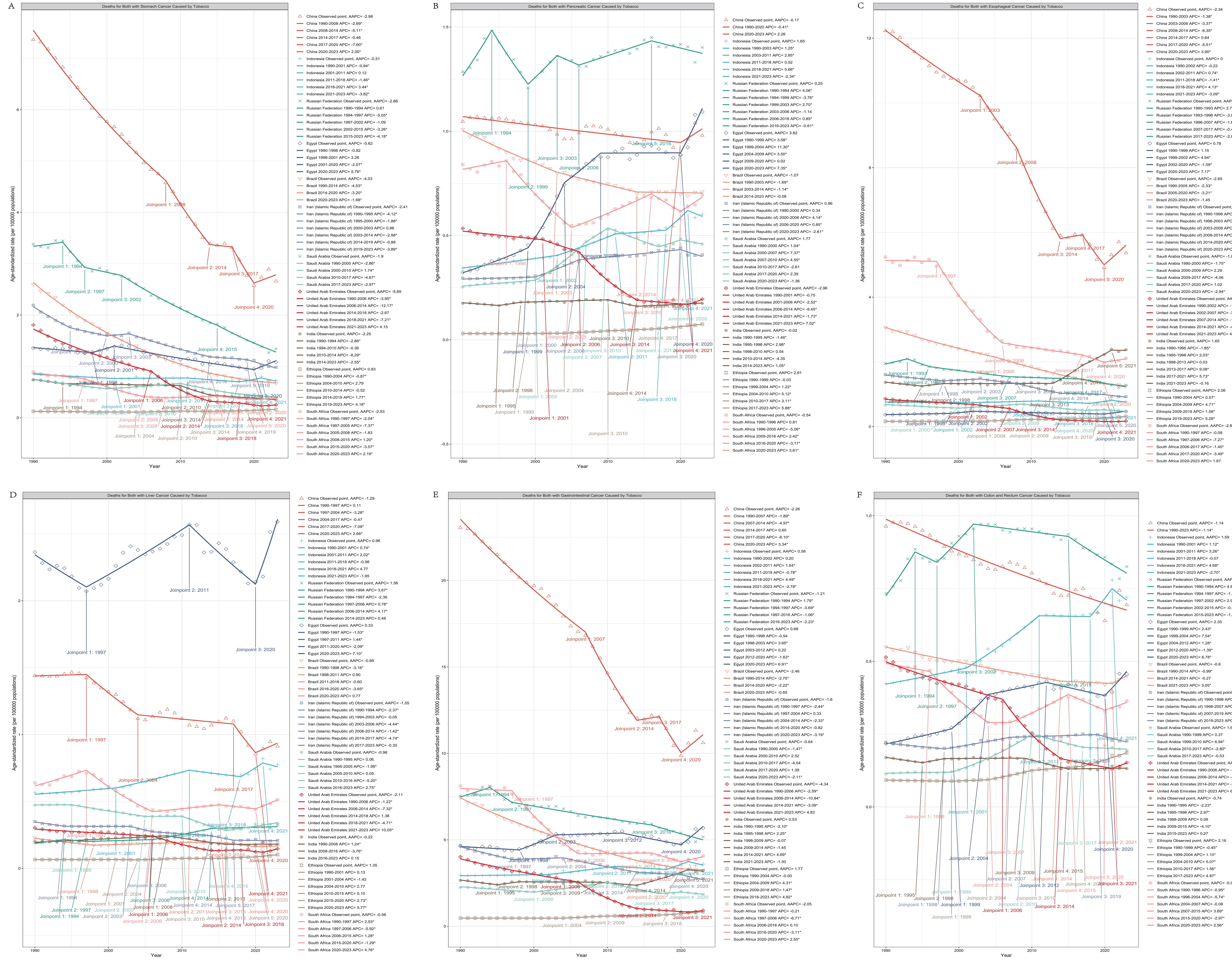

BRICS: Brazil, Russia, India, China, South Africa
APC (%/year): Annual Percentage Change
AAPC (%/year): Average Annual Percentage Change

Supplementary file Figure 3 Disparities of Age-Standardized DALYs Rates of Gastrointestinal Cancers in BRICS Countries (2023)

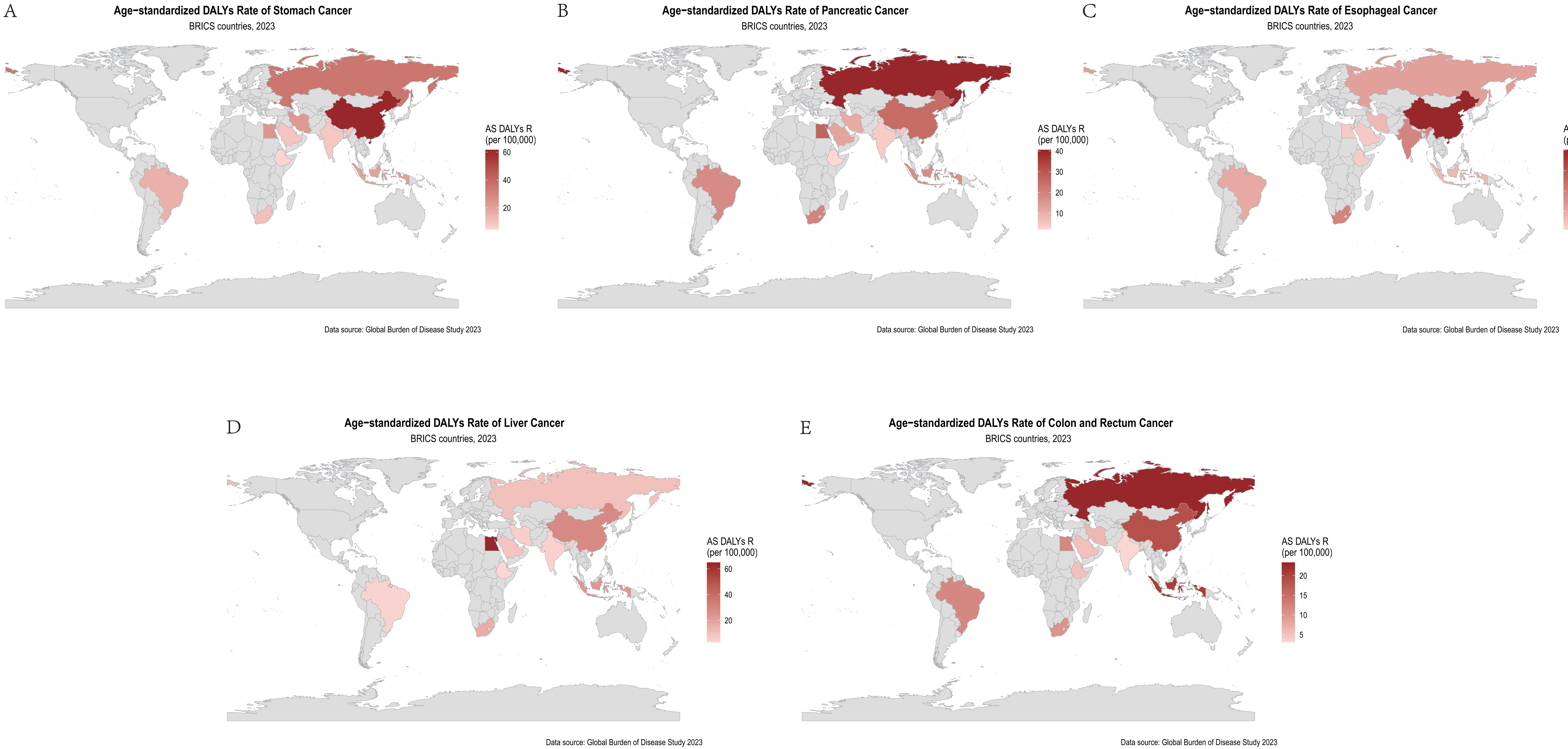

DALYs: Disability-Adjusted Life Years
BRICS: Brazil, Russia, India, China, and South Africa
AS DALYs R: Age-Standardized DALYs Rate
Cancer types: stomach cancer, pancreatic cancer, esophageal cancer,liver cancer, and colorectal cancer are the five main gastrointestinal cancer subtypes attributable to smoking.

Supplementary file Figure 4 Tobacco-Related Gastrointestinal Cancer DALYs Dynamics in BRICS (1990-2023)

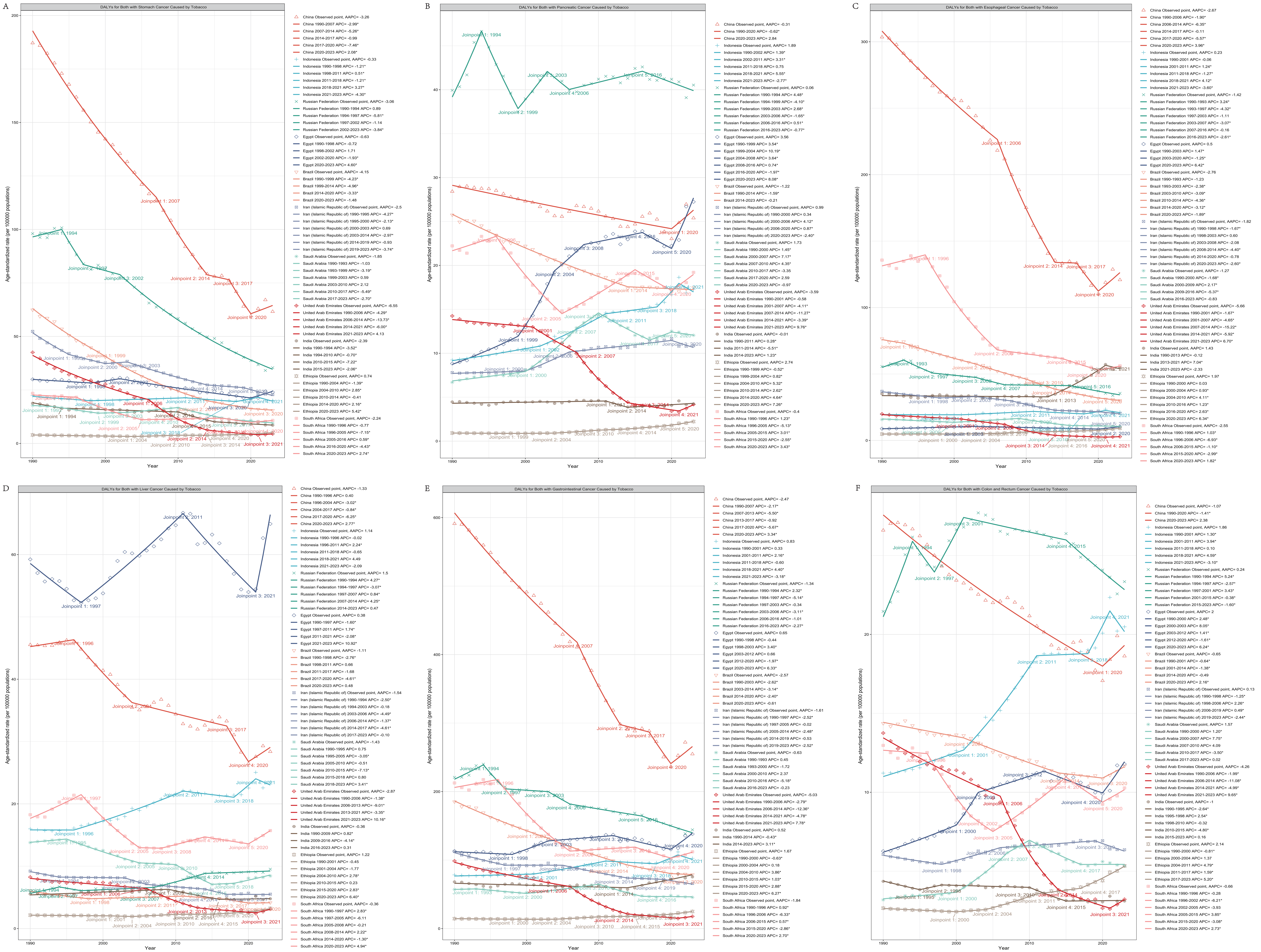

BRICS: Brazil, Russia, India, China, South Africa
DALYs: Disability-Adjusted Life Years
APC (%/year): Annual Percentage Change
AAPC (%/year): Average Annual Percentage Change

Supplementary file Table 1. Mortality burden of smoking-attributable gastrointestinal cancers among BRICS and Associated Economies in 1990: Estimates from the Global Burden of Disease (GBD) 2023 study

	Brazil	China	Egypt	Ethiopia	India	Indonesia	Iran (Islamic Republic of)	Russian Federation	Saudi Arabia	South Africa	United Arab Emirates	BRICS
	**1990**	**1990**	**1990**	**1990**	**1990**	**1990**	**1990**	**1990**	**1990**	**1990**	**1990**	**1990**
**Gastrointestinal Cancer**												
Number of cases	6495.61 (4904.00, 8171.55)	196546.26 (134964.81, 266308.90)	1176.12 (499.60, 2131.38)	105.71 (45.59, 202.72)	11909.74 (7116.51, 17763.03)	2926.33 (1552.87, 4766.64)	1196.00 (644.86, 1999.47)	14140.38 (10562.46, 18294.90)	127.18 (63.55, 225.36)	1665.39 (1029.00, 2445.91)	16.35 (7.36, 30.74)	236305.08 (161390.63, 322340.60)
All ages death rate (per 100,000	4.35 (3.28, 5.47)	16.67 (11.45, 22.59)	2.12 (0.90, 3.84)	0.21 (0.09, 0.41)	1.41 (0.84, 2.10)	1.58 (0.84, 2.57)	2.09 (1.13, 3.50)	9.32 (6.96, 12.05)	0.78 (0.39, 1.39)	4.47 (2.76, 6.56)	0.87 (0.39, 1.63)	43.87 (29.03, 62.12)
Age-standardised death rate (per 100,000)	7.44 (5.59, 9.42)	23.05 (15.82, 31.36)	4.63 (1.96, 8.49)	0.47 (0.21, 0.91)	2.65 (1.57, 3.95)	2.95 (1.58, 4.80)	4.67 (2.51, 7.80)	7.54 (5.62, 9.76)	2.24 (1.13, 3.98)	8.11 (4.96, 11.90)	4.02 (1.80, 7.55)	67.78 (42.76, 99.90)
**Esophageal_cancer**												
Number of cases	2669.52 (2046.97, 3265.31)	103028.41 (73677.40, 129072.94)	91.25 (61.21, 129.95)	35.11 (18.37, 60.39)	6116.65 (3979.80, 8462.89)	738.94 (458.01, 1085.32)	397.37 (236.37, 628.49)	3685.50 (2817.93, 4611.08)	34.63 (19.85, 58.01)	1068.37 (701.28, 1496.26)	3.06 (1.29, 5.16)	117868.82 (84018.47, 148875.81)
All ages death rate (per 100,000)	1.79 (1.37, 2.19)	8.74 (6.25, 10.95)	0.16 (0.11, 0.23)	0.07 (0.04, 0.12)	0.72 (0.47, 1.00)	0.40 (0.25, 0.58)	0.70 (0.41, 1.10)	2.43 (1.86, 3.04)	0.21 (0.12, 0.36)	2.87 (1.88, 4.02)	0.16 (0.07, 0.27)	18.25 (12.83, 23.86)
Age-standardised death rate (per 100,000)	3.06 (2.33, 3.75)	12.23 (8.72, 15.44)	0.37 (0.24, 0.52)	0.16 (0.09, 0.27)	1.37 (0.88, 1.88)	0.76 (0.47, 1.11)	1.61 (0.94, 2.53)	1.95 (1.48, 2.44)	0.67 (0.38, 1.14)	5.24 (3.38, 7.29)	0.86 (0.36, 1.43)	28.26 (19.29, 37.81)
**Stomach_cancer**												
Number of cases	2232.23 (1739.62, 2760.99)	62622.44 (44992.24, 91485.20)	312.18 (159.82, 491.80)	28.73 (10.76, 55.47)	3287.40 (1932.91, 5135.32)	836.31 (444.77, 1291.49)	560.16 (303.46, 915.84)	6328.92 (4890.30, 8110.49)	39.55 (22.64, 65.92)	184.07 (114.88, 272.14)	6.76 (3.21, 12.86)	76438.76 (54614.62, 110597.53)
All ages death rate (per 100,000)	1.49 (1.16, 1.85)	5.31 (3.82, 7.76)	0.56 (0.29, 0.89)	0.06 (0.02, 0.11)	0.39 (0.23, 0.61)	0.45 (0.24, 0.70)	0.98 (0.53, 1.60)	4.17 (3.22, 5.34)	0.24 (0.14, 0.41)	0.49 (0.31, 0.73)	0.36 (0.17, 0.68)	14.51 (10.13, 20.68)
Age-standardised death rate (per 100,000)	2.60 (2.01, 3.25)	7.37 (5.27, 10.81)	1.36 (0.69, 2.16)	0.12 (0.05, 0.24)	0.74 (0.43, 1.16)	0.86 (0.46, 1.32)	2.19 (1.18, 3.62)	3.38 (2.61, 4.33)	0.74 (0.41, 1.23)	0.90 (0.56, 1.34)	1.81 (0.86, 3.39)	22.05 (14.52, 32.86)
**Pancreatic_cancer**												
Number of cases	895.94 (759.62, 1054.07)	9148.02 (7186.83, 11412.95)	91.00 (65.66, 128.06)	6.76 (3.66, 12.02)	785.12 (523.98, 1090.60)	345.06 (239.17, 463.15)	80.95 (52.32, 129.80)	2401.27 (1931.47, 2946.32)	14.95 (9.80, 23.04)	173.47 (123.79, 235.79)	2.55 (1.45, 4.52)	13945.08 (10897.75, 17500.32)
All ages death rate (per 100,000)	0.60 (0.51, 0.71)	0.78 (0.61, 0.97)	0.16 (0.12, 0.23)	0.01 (0.01, 0.02)	0.09 (0.06, 0.13)	0.19 (0.13, 0.25)	0.14 (0.09, 0.23)	1.58 (1.27, 1.94)	0.09 (0.06, 0.14)	0.47 (0.33, 0.63)	0.14 (0.08, 0.24)	4.25 (3.27, 5.49)
Age-standardised death rate (per 100,000)	1.01 (0.85, 1.19)	1.05 (0.82, 1.30)	0.32 (0.23, 0.46)	0.03 (0.02, 0.05)	0.17 (0.12, 0.24)	0.34 (0.24, 0.46)	0.30 (0.19, 0.48)	1.28 (1.03, 1.58)	0.25 (0.17, 0.40)	0.84 (0.60, 1.16)	0.53 (0.29, 0.97)	6.14 (4.55, 8.30)
**Liver_cancer**												
Number of cases	207.82 (63.35, 358.60)	13428.79 (4297.81, 22381.26)	618.53 (178.96, 1279.49)	15.52 (3.99, 38.92)	962.77 (294.13, 1829.82)	589.73 (189.78, 1254.25)	96.33 (22.70, 209.00)	355.18 (117.59, 592.00)	30.87 (7.61, 65.36)	137.04 (35.26, 281.80)	1.47 (0.36, 3.11)	16444.06 (5211.55, 28293.62)
All ages death rate (per 100,000)	0.14 (0.04, 0.24)	1.14 (0.36, 1.90)	1.12 (0.32, 2.31)	0.03 (0.01, 0.08)	0.11 (0.03, 0.22)	0.32 (0.10, 0.68)	0.17 (0.04, 0.37)	0.23 (0.08, 0.39)	0.19 (0.05, 0.40)	0.37 (0.09, 0.76)	0.08 (0.02, 0.17)	3.90 (1.15, 7.50)
Age-standardised death rate (per 100,000)	0.23 (0.07, 0.40)	1.44 (0.46, 2.41)	2.36 (0.69, 4.97)	0.07 (0.02, 0.16)	0.20 (0.06, 0.38)	0.57 (0.18, 1.21)	0.35 (0.08, 0.76)	0.19 (0.06, 0.32)	0.47 (0.12, 1.01)	0.64 (0.16, 1.32)	0.30 (0.08, 0.67)	6.82 (1.98, 13.60)
**Colorectal_cancer**												
Number of cases	490.11 (294.45, 732.58)	8318.60 (4810.53, 11956.54)	63.15 (33.95, 102.08)	19.58 (8.81, 35.92)	757.80 (385.69, 1244.39)	416.29 (221.14, 672.43)	61.20 (30.00, 116.34)	1369.50 (805.17, 2035.01)	7.17 (3.65, 13.04)	102.45 (53.79, 159.91)	2.51 (1.05, 5.09)	11608.36 (6648.24, 17073.32)
All ages death rate (per 100,000)	0.33 (0.20, 0.49)	0.71 (0.41, 1.01)	0.11 (0.06, 0.18)	0.04 (0.02, 0.07)	0.09 (0.05, 0.15)	0.22 (0.12, 0.36)	0.11 (0.05, 0.20)	0.90 (0.53, 1.34)	0.04 (0.02, 0.08)	0.27 (0.14, 0.43)	0.13 (0.06, 0.27)	2.96 (1.65, 4.59)
Age-standardised death rate (per 100,000)	0.55 (0.33, 0.83)	0.96 (0.56, 1.39)	0.22 (0.12, 0.37)	0.09 (0.04, 0.17)	0.17 (0.09, 0.28)	0.42 (0.23, 0.69)	0.22 (0.11, 0.42)	0.73 (0.43, 1.09)	0.11 (0.06, 0.20)	0.50 (0.26, 0.80)	0.51 (0.21, 1.09)	4.50 (2.43, 7.33)

ASDR: Age-Standardized Death Rate

Values in parentheses: 95% Uncertainty Intervals (UI) for corresponding estimates

Cancer types: Esophageal cancer, stomach cancer, pancreatic cancer, liver cancer, and colorectal cancer are the five main gastrointestinal cancer subtypes attributable to smoking.

Population: BRICS and associated economies including Brazil, China, Egypt, Ethiopia, India, Indonesia, Iran, Russian Federation, Saudi Arabia, South Africa, and United Arab Emirates.

Supplementary file Table 2. DALYs burden of smoking-attributable gastrointestinal cancers among BRICS and Associated Economies in 1990: Estimates from the Global Burden of Disease (GBD) 2023 study

	Brazil	China	Egypt	Ethiopia	India	Indonesia	Iran (Islamic Republic of)	Russian Federation	Saudi Arabia	South Africa	United Arab Emirates	BRICS
	1990	1990	1990	1990	1990	1990	1990	1990	1990	1990	1990	1990
**Gastrointestinal_Cancer**												
Number of cases	174796.46 (132571.00, 218394.20)	5387125.61 (3700499.32,7317550.99)	33305.19 (14118.44 59747.23)	3550.40 (1513.78,6838.40)	345371.44 (206682.67,514965.00)	86234.57 (45864.30, 141360.63)	31978.17 (17337.80, 53827.61)	414263.35 (312169.37, 533685.29)	3772.85 (1844.56, 6705.20)	46626.51 (28766.60, 67900.72)	518.39 (232.19, 980.45)	6527542.93 (4461600.02, 8921955.71)
All ages DALYs rate (per 100,000)	117.05 (88.78, 146.25)	456.87 (313.83, 620.58)	60.08 (25.47, 107.77)	7.17 (3.06, 13.81)	40.86 (24.45, 60.92)	46.44 (24.70, 76.12)	55.99 (30.36, 94.25)	272.94 (205.68, 351.62)	23.22 (11.35, 41.27)	125.12 (77.19, 182.21)	27.54 (12.33, 52.08)	1233.27 (817.19, 1746.89)
Age-standardised DALYs rate per 100,000	185.23 (139.88, 232.44)	591.16 (405.51, 802.16)	113.13 (48.06, 204.55)	14.49 (6.24, 27.74)	68.21 (40.85, 101.78)	78.42 (41.73, 127.80)	112.47 (60.79, 188.33)	220.85 (166.47, 284.63)	57.12 (28.56, 100.86)	212.57 (131.76, 310.00)	98.70 (44.17, 185.54)	1752.36 (1114.03, 2565.83)
**Esophageal_cancer**												
Number of cases	72036.00 (55356.93, 87821.05)	2726319.14 (1968305.05, 3412089.46)	2597.42 (1704.08, 3701.74)	1150.13 (600.04, 1950.28)	177807.88 (116166.50, 245672.02)	21114.83 (13140.00, 31399.08)	10252.26 (6153.64, 16237.86)	105755.54 (81349.32, 131636.63)	925.07 (528.65, 1526.20)	29515.36 (19583.60, 40920.28)	89.47 (37.58, 153.59)	3147563.08 (2262925.39, 3973108.20)
All ages DALYs rate (per 100,000)	48.24 (37.07, 58.81)	231.21 (166.93, 289.37)	4.69 (3.07, 6.68)	2.32 (1.21, 3.94)	21.03 (13.74, 29.06)	11.37 (7.08, 16.91)	17.95 (10.78, 28.43)	69.68 (53.60, 86.73)	5.69 (3.25, 9.39)	79.20 (52.55, 109.81)	4.75 (2.00, 8.16)	496.14 (351.27, 647.29)
Age-standardised DALYs rate per 100,000	76.57 (58.55, 93.78)	303.49 (218.01, 378.97)	8.84 (5.91, 12.63)	4.81 (2.51, 8.22)	35.07 (22.85, 48.58)	19.64 (12.23, 28.78)	37.01 (22.02, 58.49)	55.65 (42.66, 69.36)	15.72 (9.03, 26.29)	135.71 (89.98, 188.69)	19.91 (8.26, 32.92)	712.43 (492.03, 946.72)
**Stomach_cancer**												
Number of cases	58310.90 (46013.51, 71390.85)	1700316.80 (1234236.39, 2471722.41)	8212.33 (4193.44, 12621.57)	1001.59 (377.09, 1937.49)	94387.51 (55549.60, 147365.44)	24303.17 (13085.86, 37551.87)	14824.44 (8161.48, 24439.10)	183545.12 (143567.41, 234477.26)	1105.34 (633.68, 1867.36)	5178.61 (3124.50, 7385.82)	203.22 (95.04, 388.31)	2091389.04 (1509038.00, 3011147.48)
All ages DALYs rate (per 100,000)	39.05 (30.81, 47.81)	144.20 (104.67, 209.62)	14.81 (7.56, 22.77)	2.02 (0.76, 3.91)	11.17 (6.57, 17.43)	13.09 (7.05, 20.22)	25.96 (14.29, 42.79)	120.93 (94.59, 154.49)	6.80 (3.90, 11.49)	13.90 (8.38, 19.82)	10.79 (5.05, 20.63)	402.72 (283.64, 570.98)
Age-standardised DALYs rate per 100,000	62.42 (48.86, 76.83)	187.13 (135.00, 273.62)	29.96 (15.28, 47.05)	3.96 (1.49, 7.63)	18.79 (11.10, 29.38)	22.33 (11.94, 34.47)	52.37 (28.59, 85.93)	98.06 (76.91, 125.27)	17.73 (10.13, 29.40)	23.42 (14.37, 33.79)	42.54 (20.12, 80.89)	558.71 (373.78, 824.27)
**Pancreatic_cancer**												
Number of cases	24560.89 (21015.41, 28636.61)	265123.45 (209109.90, 335055.24)	2798.20 (2011.03, 3947.29)	224.95 (122.22, 404.15)	22328.31 (14912.96, 30833.59)	10227.97 (6998.70, 13741.57)	2322.42 (1485.35, 3676.80)	74144.68 (60026.45, 90281.41)	461.48 (299.31, 699.67)	4885.87 (3433.19, 6571.25)	86.80 (49.88, 151.02)	407165.04 (319464.37, 513998.62)
All ages DALYs rate (per 100,000)	16.45 (14.07, 19.18)	22.48 (17.73, 28.42)	5.05 (3.63, 7.12)	0.45 (0.25, 0.82)	2.64 (1.76, 3.65)	5.51 (3.77, 7.40)	4.07 (2.60, 6.44)	48.85 (39.55, 59.48)	2.84 (1.84, 4.31)	13.11 (9.21, 17.63)	4.61 (2.65, 8.02)	126.06 (97.07, 162.46)
1990–2023 AAPC in AS DALYs R (% per year)	-1.22 (-1.27 to -1.16)	-0.31 (-0.53 to -0.16)	3.56 (3.43 to 3.68)	2.74 (2.67 to 2.8)	-0.01 (-0.14 to 0.14)	1.89 (1.79 to 1.99)	0.99 (0.86 to 1.09)	0.06 (-0.02 to 0.14)	1.73 (1.63 to 1.82)	-0.4 (-0.52 to -0.29)	-3.59 (-3.74 to -3.42)	0.24 (0.19 to 0.29)
**Liver_cancer**												
Number of cases	5984.15 (1859.08, 10063.43)	446242.67 (144517.59, 744730.79)	17616.84 (5105.71, 36082.63)	549.22 (140.26, 1361.44)	28942.61 (8959.38, 55206.69)	18301.01 (6043.55, 38918.92)	2753.69 (646.56, 6041.33)	10738.44 (3586.46, 17939.96)	1036.75 (258.38, 2177.44)	4204.43 (1089.62, 8515.83)	50.60 (12.13, 104.58)	536420.40 (172218.73, 921143.05)
All ages DALYs rate (per 100,000)	4.01 (1.24, 6.74)	37.84 (12.26, 63.16)	31.78 (9.21, 65.09)	1.11 (0.28, 2.75)	3.42 (1.06, 6.53)	9.86 (3.25, 20.96)	4.82 (1.13, 10.58)	7.08 (2.36, 11.82)	6.38 (1.59, 13.40)	11.28 (2.92, 22.85)	2.69 (0.64, 5.56)	120.27 (35.96, 229.43)
Age-standardised DALYs rate per 100,000	6.13 (1.89, 10.40)	45.52 (14.71, 75.85)	59.24 (17.18, 122.35)	2.15 (0.55, 5.36)	5.53 (1.70, 10.50)	15.99 (5.19, 34.11)	9.22 (2.17, 20.05)	5.74 (1.92, 9.60)	13.79 (3.41, 29.04)	18.26 (4.72, 37.12)	8.25 (2.07, 17.78)	189.82 (55.51, 372.16)
**Colorectal_cancer**												
Number of cases	13904.53 (8326.07, 20482.25)	249123.56 (144330.39, 353953.08)	2080.40 (1104.18, 3393.99)	624.51 (274.17, 1185.04)	21905.13 (11094.22, 35887.26)	12287.58 (6596.19, 19749.19)	1825.35 (890.76, 3432.51)	40079.57 (23639.73, 59350.02)	244.21 (124.54, 434.53)	2842.22 (1535.69, 4507.55)	88.30 (37.57, 182.95)	345005.37 (197953.52, 502558.36)
All ages DALYs rate (per 100,000)	9.31 (5.58, 13.72)	21.13 (12.24, 30.02)	3.75 (1.99, 6.12)	1.26 (0.55, 2.39)	2.59 (1.31, 4.25)	6.62 (3.55, 10.64)	3.20 (1.56, 6.01)	26.41 (15.58, 39.10)	1.50 (0.77, 2.67)	7.63 (4.12, 12.10)	4.69 (2.00, 9.72)	88.08 (49.24, 136.73)
Age-standardised DALYs rate per 100,000	14.36 (8.62, 21.26)	26.60 (15.38, 37.95)	6.27 (3.34, 10.10)	2.66 (1.19, 4.89)	4.36 (2.22, 7.13)	11.22 (5.97, 18.06)	6.07 (2.97, 11.45)	21.46 (12.67, 31.77)	3.19 (1.62, 5.77)	12.97 (6.95, 20.49)	13.75 (5.77, 28.33)	122.89 (66.71, 197.20)

DALYs: Disability-Adjusted Life Years
AS DALYs R: Age-Standardised DALYs Rate
Values in parentheses: 95% Uncertainty Intervals (UI) for corresponding estimates

Cancer types: Esophageal cancer, stomach cancer, pancreatic cancer, liver cancer, and colorectal cancer are the five main gastrointestinal cancer subtypes attributable to smoking.
Population: BRICS and associated economies including Brazil, China, Egypt, Ethiopia, India, Indonesia, Iran (Islamic Republic of), Russian Federation, Saudi Arabia, South Africa, and United Arab Emirates.
